# Fatal Necrotizing Encephalopathy after Treatment with Nivolumab for Squamous Non-Small Cell Lung Cancer: Case Report and Review of the Literature

**DOI:** 10.3389/fimmu.2018.00108

**Published:** 2018-01-30

**Authors:** Markus Leitinger, Mihael V. Varosanec, Slaven Pikija, Romana E. Wass, Dave Bandke, Serge Weis, Michael Studnicka, Susanne Grinzinger, Mark R. McCoy, Larissa Hauer, Johann Sellner

**Affiliations:** ^1^Department of Neurology, Christian Doppler Medical Center, Paracelsus Medical University, Salzburg, Austria; ^2^Department of Pulmonary Medicine, University Hospital Salzburg, Paracelsus Medical University, Salzburg, Austria; ^3^Division of Neuropathology, Institute of Pathology and Microbiology, Neuromed Campus-Kepler University Hospital, Linz, Austria; ^4^Division of Neuroradiology, Christian Doppler Medical Center, Paracelsus Medical University Salzburg, Salzburg, Austria; ^5^Department of Psychiatry and Psychotherapy, Christian Doppler Medical Center, Paracelsus Medical University Salzburg, Salzburg, Austria; ^6^Department of Neurology, Klinikum rechts der Isar, Technische Universität München, München, Germany

**Keywords:** neuroinflammation, neurodegeneration, encephalitis, immune checkpoint inhibitors, nivolumab, humoral and cellular immune response, autoreactive antibodies

## Abstract

Immune checkpoint inhibitors are antibodies, which enhance cellular and humoral immune responses and are approved for the treatment of various tumors. Immune-related adverse events (irAE) involving different organs and systems are, however, among the side-effects. Recent reports of severe persistent neurological deficits and even fatal cases underpin the need for better understanding of the exact pathomechanisms of central nervous system (CNS) toxicity. To our knowledge, we report the first biopsy-proven case of fatal necrotizing encephalopathy after treatment with nivolumab. Nivolumab targets the immune-check point inhibitor programmed cell death-1 and was used for squamous non-small cell lung cancer. Partly reversible neurologic and psychiatric symptoms and unremarkable brain magnetic resonance imaging (MRI) were observed after the first course. Neurological symptoms progressed and recurrent seizures developed after the second course. Brain MRI disclosed multiple edematous and confluent supra- and infratentorial lesions, partly with contrast-enhancement. We excluded autoimmune and paraneoplastic causes and performed ancillary investigations to rule out common and opportunistic infections. Eventually, postmortem histopathological analysis of the brain revealed a necrotizing process, which contrasts previous cases reporting parenchymal immune cell infiltration or demyelination. Appropriate diagnostic pathways and treatment algorithms need to be implemented for the work-up of CNS toxicity and irAEs related to immune checkpoint inhibitor treatment.

## Introduction

The immune system has a relevant role in anticancer response (1). Immune checkpoints including cytotoxic T-lymphocyte-associated antigen 4 (CTLA-4) and programmed cell death-1 (PD-1) are receptors on the surface of activated CD8 positive T cells, which act as brakes for such immune response (2). The physiological rationale for the interaction of these receptors with their ligands is based on the necessity to promote self-tolerance and thus prevent autoimmunity (3). Immune checkpoint inhibitors are antibodies, which target these regulatory receptors and enhance pre-existing immune responses. Ipilimumab, an inhibitor of CTLA-4, is approved for the treatment of advanced or unresectable melanoma. Nivolumab and pembrolizumab, both PD-1 inhibitors, are used for the treatment of advanced or metastatic melanoma and patients with metastatic, refractory non-small cell lung cancer (NSCLC). In addition, the combination of ipilimumab and nivolumab has been approved in patients with wild-type v-Raf murine sarcoma viral oncogene homolog B (BRAF) metastatic or unresectable melanoma (4).

Lung cancer is the leading cause of cancer death worldwide (5). NSCLC accounts for 85% of the cases of lung cancer and diagnosis is predominantly made when the disease is advanced or metastatic. Nivolumab is a human IgG4 anti-PD-1 monoclonal antibody (6, 7). In the pivotal Checkmate 057 trial, nivolumab achieved improved outcomes in terms of response and survival over standard chemotherapy (8). Further approvals include advanced renal cell carcinoma after prior therapy in adults and relapsed or refractory classical Hodgkin lymphoma after autologous stem cell transplant and treatment with brentuximab vedotin (9, 10). Additional indications are squamous cell cancer of the head and neck in adults progressing on or after platinum-based therapy and locally advanced unresectable or metastatic urothelial carcinoma in adults after failure of prior platinum-containing therapy (11, 12). Furthermore, pembrolizumab demonstrated superiority over standard first line platinum-based chemotherapy in patients with advanced or metastatic NSCLC with PDL-1 expression >50% (13). Thus, these approvals provide a ray of hope in the management of these and other neoplastic conditions.

The clinical benefits of these treatments are, however, overshadowed by the associated toxicities (14, 15). Since immune checkpoint inhibition results in impaired self-tolerance and exacerbation and even *de novo* development of autoimmune reactions, patients with pre-existing autoimmune disorders were excluded from clinical trials. Still, immune-related adverse events (irAEs) distinct from side-effects observed with conventional cytotoxic chemotherapy. They arise from systemic inflammation and included dermatologic, gastrointestinal, hepatic, respiratory, renal, and endocrine manifestations (16). In this regard, transverse myelitis, meningitis, posterior reversible encephalopathy syndrome (PRES), and limbic encephalitis were observed in the clinical trials of nivolumab (Opdivo^®^, Bristol-Myers-Squibb, New York, NY, USA) (17). Cases of detrimental and fatal irAEs of the central nervous system (CNS) in the post-marketing phase such as immune-mediated encephalitis and myelitis sparked further interest in these conditions (18–23). There is insufficient understanding of the pathomechanisms leading to CNS toxicity and subsequent management (24). Thus, the U.S. Food and Drug Administration issued an ongoing post-marketing requirement for enhanced pharmacovigilance to evaluate incidence, severity and outcomes.

Here, we expand the spectrum of checkpoint inhibitor-related toxicity to the CNS by reporting a fatal and histologically proven case of necrotizing encephalopathy after two cycles of nivolumab as second-line treatment for squamous NSCLC.

## Case Presentation

**Figure 1:**
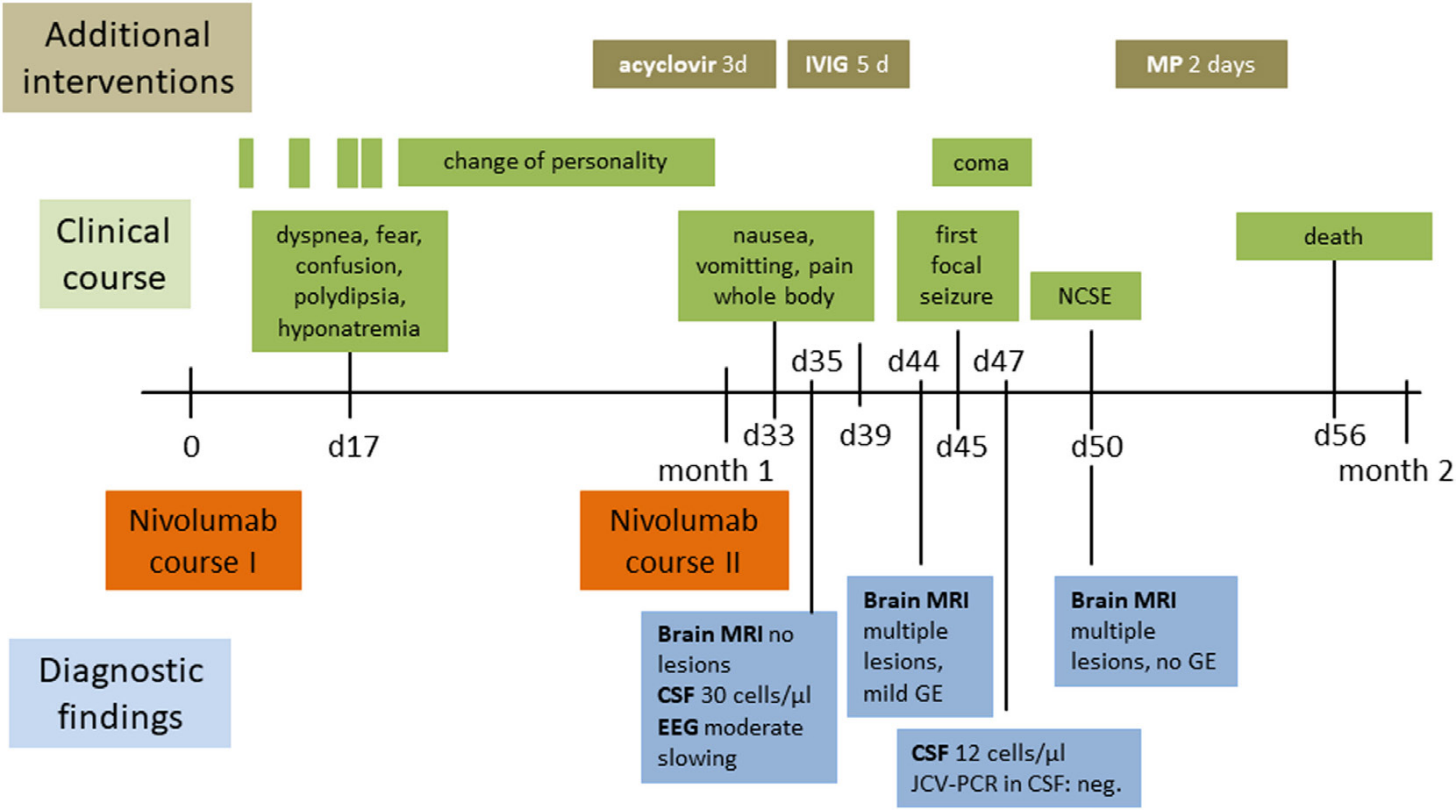
Clinical, therapeutic, and radiological course. Abbreviations: CSF cerebrospinal fluid; d, days; EEG, electroencephalography; GE, gadolinium-enhancement; IVIG, intravenous immunoglobulin; JCV-PCR John Cunningham virus-polymerase chain reaction; MP, methylprednisolone; MRI, magnetic resonance imaging; NCSE, non-convulsive status epilepticus.

**Figure 2:**
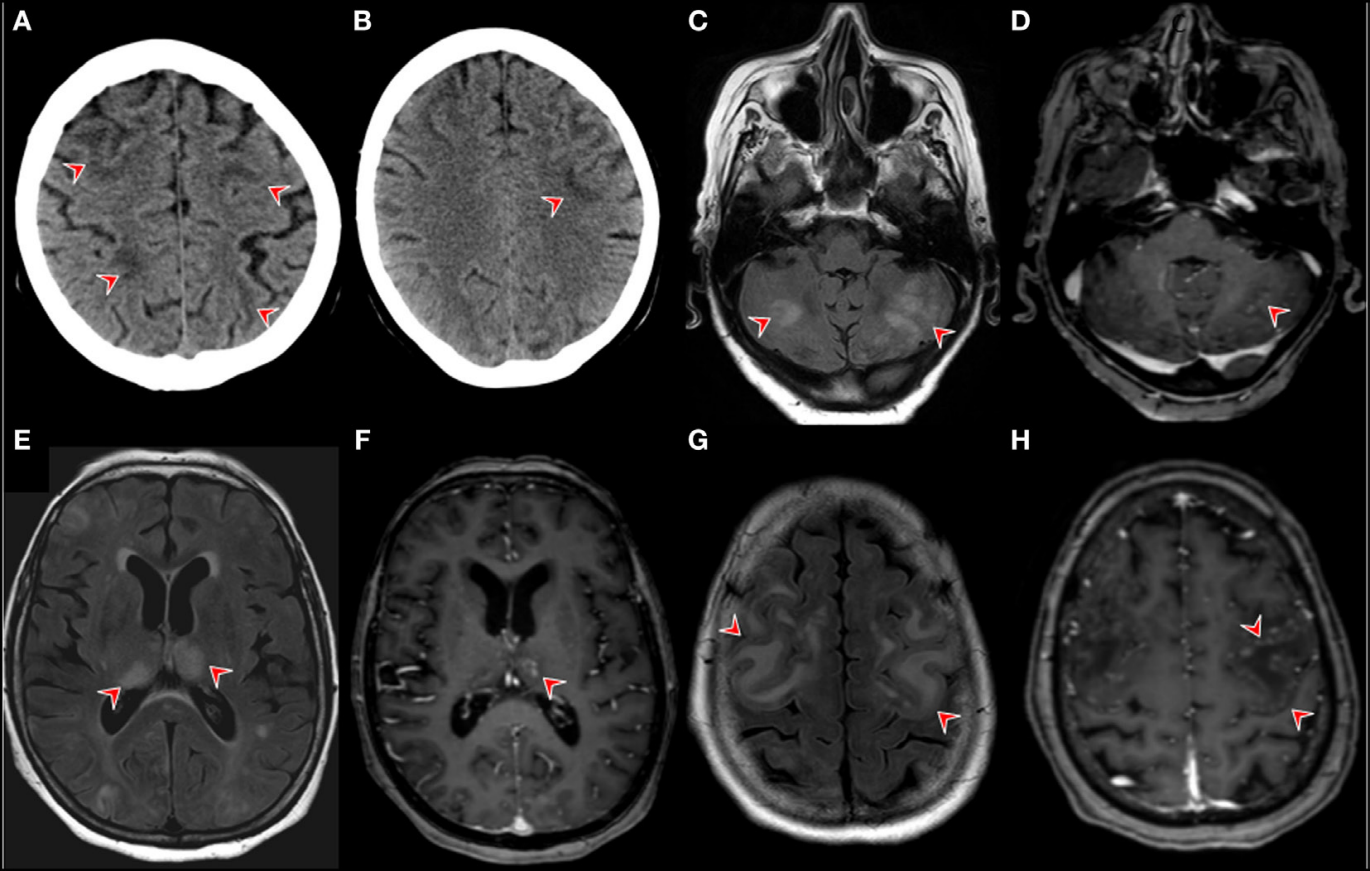
Neuroimaging. Brain CT in the postoperative course after the patient developed nausea, vomiting, and generalized weakness. The red arrows point at revealing wide-spread bilateral hypodensities in the subcortical white matter of the frontal, parietal, and occipital lobe **(A,B)**. Brain MRI findings on day 14 of month 1 of the first nivolimab course. Fluid-attenuated inversion recovery (FLAIR) magnetic resonance imaging (MRI) showing multiple bilateral hyperintensities in gray cerebellar matter [**(C)**, red arrows]. **(D)** T1-contrast enhanced images on the same level as image [**(A)** (red arrow)]. **(D)** MRI FLAIR images showing bilateral thalamic hyperintensities with corresponding T1-contrast enhancement left [**(F)**, red arrow]. FLAIR MRI images showing confluent cortical hyperintensities **(G)** T1 contrast-enhancement showing pial gyriform pattern of enhancement [**(H)**, red arrows].

A 67-year-old woman was diagnosed with squamous NSCLC 1 year before the current admission, details of the subsequent clinical course are outlined in Figure 1. The work-up including PET/CT and analysis of the specimen removed by partial resection of the lower lobe of the right lung, pleura, and specimens of the sixth rib staged the tumor as pT3; pN0 (0/14); L0, V0; G2-G3; R0. Further immunohistological analyses showed the following reactivities: CK-5/6 (+), ALK D5-F3 (−), c-MET (++ to +++), PD-L1, and PD-1 (−), PI3K (−). Her comorbidities included hypertension, chronic renal insufficiency, recurrent hyponatremia, hypercholesterinemia, peripheral arterial occlusive disease, depression/anxiety disorder, and smoking (25 pack years). She developed nausea, vomiting, and generalized weakness in the postoperative course and was treated for hypertension and hyponatremia. Brain CT revealed wide-spread bilateral hypodense lesion in the subcortical white matter of the frontal, parietal, and occipital lobe (Figures 2A,B), which had vanished on follow-up 8 days later. Our patient recovered within a few days, and the episode was classified as reversible encephalopathy syndrome. The subsequent 24 h blood pressure monitoring revealed mean systolic day- and nighttime blood pressure of 135 and 142 mmHg, respectively.

Our patient was started on adjuvant chemotherapy with monthly carboplatin and gemcitabine. The dosage had to be reduced to 75% for the fourth and last cycle due to anemia. Examination with PET/CT 3 months before the actual admission revealed recurrence with wide-spread pleural affection. Second-line treatment with nivolumab in the standard dosage of 3 mg/kg was subsequently initiated and details of the course are shown in Figure 1. Our patient was admitted to the emergency room on day 17 after the first dose of nivolumab for dyspnea, confusion, and increased symptoms of a pre-existing anxiety disorder. The condition was attributed to hyponatremia and treatment with sertraline as a potential cause was terminated. She was released in improved condition and the second nivolumab course was given as scheduled on day 30. Three days later, she was readmitted to the hospital due to clinical deterioration with intermittent phases of disorientation and speech arrest. On neurological exam, she had fluent aphasia, perseveration, and could not execute complex requests. A complex-partial status epilepticus was ruled out by electroencephalography (EEG) at this time. The examination of cerebrospinal fluid (CSF) on day 35 after the first course of nivolumab revealed a lymphomonocytic pleocytosis of 30 cells/μl (normal <5) with a few neutrophils and macrophages, the protein level was 56 mg/dl (range 3–50). Glucose and lactate were within the limits. CSF IgG level was 6.62 mg/dl, the IgG quotient was normal, and PCR for neurotropic viruses, and oligoclonal bands were negative. Brain magnetic resonance imaging (MRI) was unremarkable and EEG showed moderate diffuse slowing. A course of antiviral treatment with acyclovir (10 mg/kg body weight, total 3 days) was initiated with the potential differential diagnosis of herpetic encephalitis. Intermittent somnolence, disorientation, and impaired communication necessitated take-over of activities of daily living by nursing staff and a nasogastric tube. We started a treatment with intravenous immunoglobulins (30 g per day equal to 0.5 g/kg bodyweight, total 5 days) upon suspicion of autoimmune encephalitis. Her condition slightly improved but, within a few days, she developed stupor. Recurrent focal seizures were observed despite antiepileptic treatment with levetiracetam (5× 500 mg). Brain MRI 44 days showed multiple and confluent cortical and subcortical FLAIR hyperintensities within both cerebral hemispheres, as shown in Figures 2C,E,G. In addition, lesions were located within both thalami, the pons and the cerebellum. A pial and gyriform contrast enhancement was observed in some lesion (Figures 2D,F,H). PCR for neurotropic viruses including HSV, CZV, and EBV were negative in CSF. Another CSF examination was performed on day 47 and revealed 12 cells/μl, protein 73 mg/dl, IgG serum/CSF quotient 7.5, and IgG 19.1 (range 0.48–5.86). Oligoclonal bands were again negative in CSF. The cells in CSF were mostly lymphocytes and monocytes, and a small number of erythrocytes and granulocytes was present. PCR was found to be negative for JCV, VZV, and HSV. Neuronal antibodies in serum (NMDA-R, AMPAR1/2, GABA-R, LGI1, CASPR2, Amphiphysin, CV2, PNMA2, Ri, Yo, Hu, Recoverin, SOX1, Titin, Zic4, GAD65, Tr) and CSF (NMDAR, AMPA-R1/2, GABA-R, LGI1, CASPR2) were negative. Follow-up brain MRI on day 50 disclosed downsized lesions and no contrast enhancement. We diagnosed non-convulsive status epilepticus in the context of episodes of reduced levels of consciousness and started anticonvulsive therapy with levetiracetam (3 × 500 mg). Seronegative autoimmune encephalitis and cerebral vasculitis were suspected and a course of methylprednisolone (1 g i.v.) was given. Steroids were stopped after 2 days of unchanged clinical condition. In a setting of comfort terminal care, the patient died on day 56 after the second course of nivolumab.

## Neuropathological Examination

**Figure 3:**
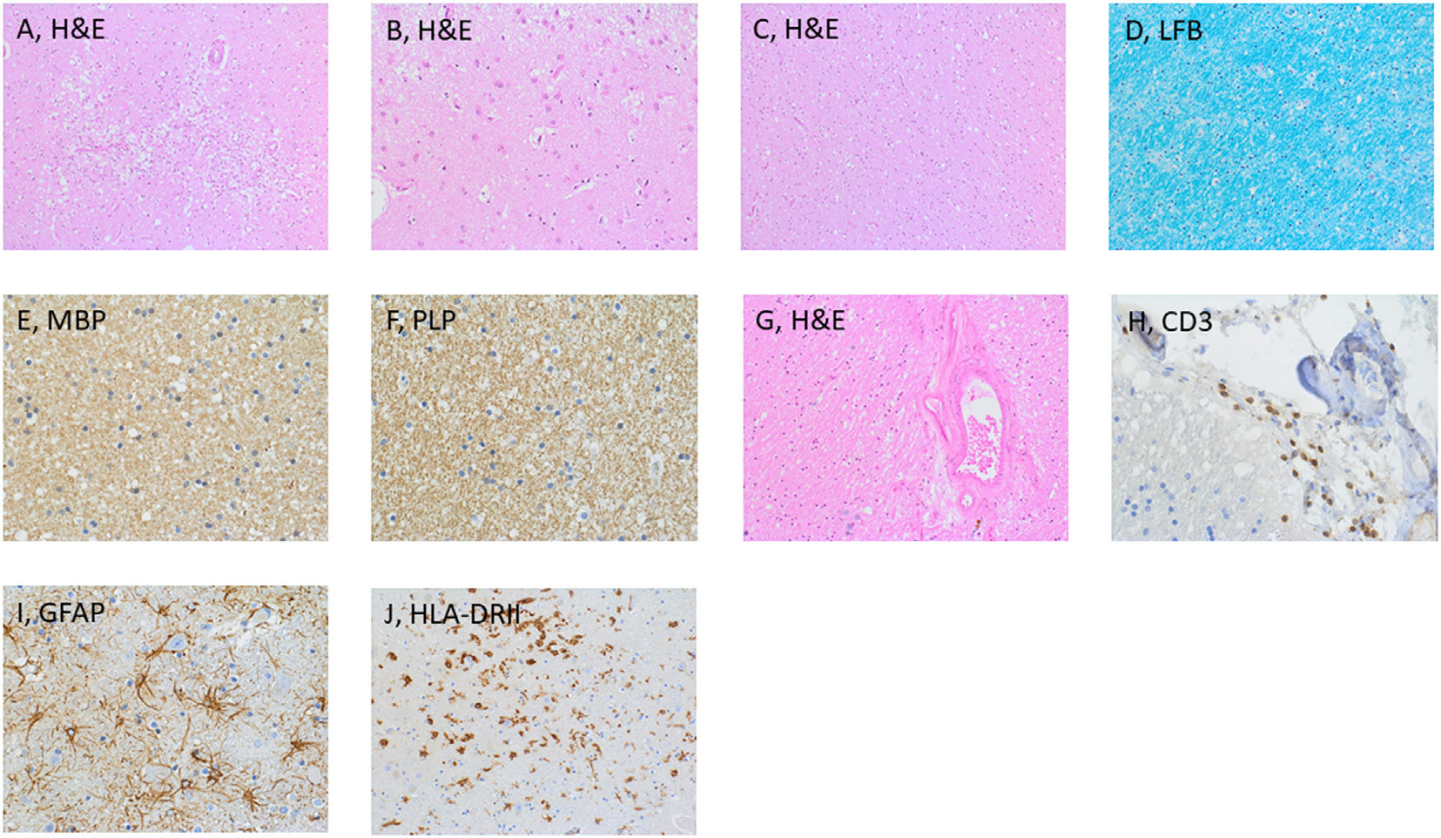
Histological examination of the brain. Microscopic examination disclosed necrotic tissue damage in the right thalamus [**(A)**, H&E], and reactive astrogliosis in the cortex [**(B)**, H&E]. Normal appearing white matter [**(C)**, H&E]. Further stainings of the thalamus included LFB **(D)**, MBP **(E)**, and PLP **(F)**. Additional findings were vascular wall hyalinosis **(G)** with presence of CD3-positive T-cells in the perivascular space **(H)**, reactive astro and microgliosis [**(I,J)**, respectively]. Abbreviations: GFAP Glial fibrillary acid protein, LFB Luxol fast blue, MBP Myelin basic protein, PLP Proteolipid protein.

The brain weight was 1,539 g. The gross-anatomic examination showed no abnormalities. Microscopic analysis revealed generalized mild edematous changes as well as hypoxic neuronal changes (Figure 3). In both thalami and the left central region, beginning tissue necrosis (stage I) was noted. The wall of gray and white matter vessels was thickened. The white matter was unremarkable with normal cell density, normal appearing myelin sheets, and no staining abnormalities for proteolipid protein, myelin basic protein, and myelin oligodendroglial glycoprotein. Rarely, a CD3-positve T-cell was found. The CD3 T-cell population consisted of CD4 and CD8 T-cells. Focally, CD3-positive T-cells were grouped around a hyalinized vessel. No presence of IgA and IgG was found, whereas IgM bound to glial cells. Reactive astro- and microgliosis was mild. No significant changes supporting an autoimmune process were noted. The neuropathological diagnosis includes necrotic tissue changes in both thalami and the left central region.

## Discussion

The spectrum of serious neurological adverse events in patients receiving immune checkpoint inhibitors is ever expanding (25). Here, we report an autopsied case of acute necrotizing encephalopathy after nivolumab treatment for NSCLC. While causality cannot be proven in our patient and nivolumab may have exacerbated a pre-existing brain disorder or susceptibility for encephalopathy, this case shares some clinical, radiological and histological features of previous cases, which were classified as nivolumab-related encephalitis and myelitis. The case is unique as clinical symptoms were partly reversible after the first nivolumab dose without steroid treatment and brain lesions could not be detected at that time point. Notably, the clinical condition deteriorated after the second dose, and then edematous disseminated lesions scattered throughout the brain and presence of contrast-enhancement appeared on MRI. While there was intrathecal inflammation as evidenced by CSF pleocytosis and increased IgG levels, IVIG and steroids did not alter the course, which contrasts most published cases of presumed CNS toxicity triggered by immune checkpoint inhibitors. Notably, histopathological analysis revealed a necrotizing process, which expands the potential spectrum of CNS toxicity of nivolumab.

A comprehensive search of the Bristol-Myers Squibb safety database, which comprises clinical trials, spontaneous, post-market literature, and solicited reports, from January 4, 2016 through July 3 2016, identified 28 encephalitis cases in association with nivolumab monotherapy or in combination with ipilimumab (26). There were 1/19 (5%) and 1/9 (11%) fatal cases of encephalitis in the mono- and combination therapy groups, respectively. Patients presented with headache, fever, weakness, tiredness, confusion, memory difficulties, sleepiness, hallucinations, seizures, and meningism (23). This confirms that the clinical presentations are not distinct from encephalitis cases of other etiology or acute encephalopathy. In addition, some encephalitis cases had seizures in the course, which were commonly refractory to anticonvulsive therapy. Many of the cases had a pleocytosis in CSF and responded to immunosuppressive and treatment discontinuation (14, 22). Immune-mediated adverse reactions are generally managed with administration of high-dose (2–4 mg/kg prednisolone equivalent) corticosteroids followed by a tapered dose and interruption of nivolumab therapy (7). Clinical outcome varies and ranges from complete resolution of neurological symptoms to fatal outcome (7). Importantly, recent reports disclosed that treatment combinations including immune checkpoint inhibitors and other antineoplastic treatments including platinum-based chemotherapy inhibitors of epidermal growth factor receptor and angiogenesis-inhibitors result in increased incidence of irAEs (27, 28). In contrast, palliative radiation therapy does not seem to increase the risk (29). Recently, Larkin et al. studied six encephalitis cases related to nivolumab or nivolumab plus ipilimumab in detail and reported a time to encephalitis of 55 days (range 18–297) (22). One case was fatal and four of five patients improved within 5–21 days on immunosuppressive therapy. The fatal case shares some features with our patient. Initial symptoms with two episodes of altered mental state were observed on day 7 after the fourth nivolumab course given for melanoma and a seizure developed on day 8. On day 10, she was readmitted for deteriorating clinical condition, developed coma within 2–3 days, and died on day 16.

Only a few cases in the literature were examined by means of histology so far, and disclosed diverse findings. There is a case of CNS demyelination after initial ipilimumab and, later, nivolumab treatment for melanoma (20). Confusion, nausea, and vomiting developed 2 days after the fourth infusion and brain MRI lesions were large and multifocal, and had mild contrast enhancement. The patient showed initial clinical and radiological improvement with IVIG but died 4 months later after progression of the brain MRI lesions. Contrasting our case where we found edema and necrosis as the underlying substrate of the lesions, there was presence of widespread demyelination in the right frontal and left temporoparietal white matter with infiltration of macrophages containing myelin debris and focal perivascular lymphoid inflammation. In the CA209-057 study, a 70-year-old female with metastatic NSCLC died due to limbic encephalitis, which developed after the 14th infusion of nivolumab (7). Again, histology revealed dense lymphocytic infiltrates in both thalami. An autopsied case of brain stem encephalitis, which occurred after ipilimumab and pembrolimumab therapy for metastatic melanoma contrasts the histopathological findings of our case as well (30). That patient had sudden lethal outcome and autopsy revealed diffuse nodular activation of microglia in the entire brain with prominent intraparenchymal and perivascular lymphocytic infiltration of the brainstem.

The histological evidence for edema and necrosis, the inflammatory CSF findings, and contrast enhancement of the lesions in our case, therefore, deserves further attention. Neutrophils were present in both CSF examinations and could reflect the necrotizing process. The detection of increased IgG levels in CSF is contrasted by lack of IgG detection within the lesions. Humoral immune responses are generated by T cell-independent and -dependent pathways. Importantly, both types of immune responses are controlled by PD-1 (31). There is high expression of PD-1 on the surface of innate-type B cells, which suppresses differentiation into long-lived plasma cells that produce IgG. Thus, the increased IgG levels might reflect an effect of nivolumab on B cells. Of note, other cases of irAE showed extensive infiltration of immune cells and thus may be pathogenetically distinct and caused by an expansion of autoreactive cytotoxic T cells. Yet, we cannot exclude that the spectrum of histopathological changes reflects a time-dependent immune process and demyelination and infiltration with immune cells develops over time (32). Moreover, the pathogenesis of CNS toxicity might be distinct for each immune checkpoint inhibitor. Our findings of extensive multifocal brain lesions and disease progression with limited response to steroids are in line with an autopsied case of ipilimumab-induced necrotic myelopathy in a patient with metastatic melanoma (33). That patient presented with acute encephalopathy and generalized tonic-seizures. MRI showed diffuse bilateral FLAIR hyperintensities involving the frontal, parietal, and occipital lobes. There were 68 cells/μl in CSF, which were predominantly lymphocytes. Four days later, that patient developed acute onset paraplegia, urinary retention, and sensory loss in the lower extremities. Spinal cord MRI visualized patchy T2-hyperintensivties with focal intramedullary lesions at C5, T3/4, T6/7, and Th11/12. Several nerve roots and cauda equina showed contrast-enhancement. Neuropathological work-up revealed necrosis accompanied by predominantly perivascular histiocytic and lymphocytic infiltrates. While acute encephalopathy in that case resolved with steroids, clinical deficits resulting from myelopathy remained unchanged. Another case of extensive spinal cord edema, which developed after two cycles of ipilimumab for metastatic melanoma may be of relevance (34). Due to a lymphocytic pleocytosis (16 cells/μl), that case was classified as myelitis but clinical response to steroids was poor. Fulminant cerebral edema necessitating emergency decompressive hemicraniectomy was reported with nivolumab for pediatric glioblastoma (35). That patient already developed hemiparesis, cerebral edema, and midline shift after the first nivolumab dose, which was managed with steroids. The condition deteriorated after the second dosage of nivolumab and the patient died despite neurosurgical intervention. Two additional encephalitis cases were reported after combined treatment with nivolumab and ipilimumab, for the treatment of melanoma and NSCLC, respectively (21). These two patients experienced clinical deterioration a few days after the first treatment cycle. Remarkably, one patient had a lesion in the right temporal lobe suggestive of limbic encephalitis; in the other patient, NMDA receptor antibodies were detected in CSF, whereas brain MRI was unremarkable. Thus, assessment of paraneoplastic disease is required in patients developing CNS symptoms in the aftermath of checkpoint inhibitor treatment.

Initial neurological symptoms in terms of acute encephalopathy developed in the postoperative phase prior to first-line therapy. CNS lesions seen on CT were reversible at that time and without the usage of steroids. The working hypothesis was reversible encephalopathy syndrome, yet, the confirmation is challenged since a brain MRI was not performed. Acute toxic encephalopathy and PRES are common in the context of cancer and treatment (36). Of note, Hussein et al. reported a case of nivolumab-induced PRES, which developed after three doses (37). This case is of interest as brain MRI lesions persisted over the observation period of 9 months. Tchapyjnikov and Borst reported a patient with nivolumab-associated Hashimoto’s thyreoiditis, PRES, and seronegative progressive encephalopathy with rigidity and myoclonus, a stiff-person-syndrome spectrum disorder (38). In contrast to our case, CSF findings were normal and full recovery could be achieved with steroids, IVIG, and plasma exchange.

Hyponatremia is a common finding in SCLC (25%) and is related to inappropriate ADH secretion (SIADH) in most of the cases (39). Hyponatremia was present in our case during the first episode of neurological symptoms. A lowering of sodium levels has also been reported in clinical trials of nivolumab, with 25% of patients experiencing some degree of hyponatremia. A rise of sodium in response to steroid treatment prompted some authors to consider an immune-mediated pathogenesis in a subgroup of patients (40). Hypophysitis can be an additional irAE of checkpoint inhibition and cause of hyponatremia (41). Since there was no evidence for involvement of the pituitary gland on MRI, ACTH and cortisol levels were not studied in our patient, and sodium levels improved over the course.

## Conclusion

The number of indications for immune checkpoint inhibitors is expanding. Durable responses and prolongation of survival with these agents come at the price of irAEs. Here, we expand the spectrum of CNS toxicity by disclosing a necrotizing process within the brain parenchyma, which contrasts previous cases reporting parenchymal immune cell infiltration or demyelination. Notably, we cannot prove causality and rule out an independent process of necrotizing encephalopathy exacerbated by nivolumab therapy. As neurologic adverse events may rapidly develop serious consequences, early recognition and management is essential. Careful differential diagnosis is mandatory. Knowledge about the presumably different pathomechanisms leading to CNS toxicity needs to be expanded.

## Ethics Statement

No investigations or interventions were performed outside routine clinical care for this patient. The husband signed an approval for the publication of the case.

## Author Contributions

ML, MV, RW, MS, and JS were involved in the clinical care of the patient and planned the case report. SG, DB and SW were responsible for the histological analysis. SP, MRM, and JS re-analyzed the MR images. ML, SW, LH, and JS wrote the first draft and created the figures. All authors reviewed and revised the manuscript and approved the final manuscript as submitted.

## Conflict of Interest Statement

The authors declare that the research was conducted in the absence of any commercial or financial relationships that could be construed as a potential conflict of interest.
